# Does Perceptual Learning Suffer from Retrograde Interference?

**DOI:** 10.1371/journal.pone.0014161

**Published:** 2010-12-07

**Authors:** Kristoffer C. Aberg, Michael H. Herzog

**Affiliations:** Laboratory of Psychophysics, Brain Mind Institute, Ecole Polytechnique Fédérale de Lausanne (EPFL), Lausanne, Switzerland; University of Regensburg, Germany

## Abstract

In motor learning, training a task B can disrupt improvements of performance of a previously learned task A, indicating that learning needs consolidation. An influential study suggested that this is the case also for visual perceptual learning [1]. Using the same paradigm, we failed to reproduce these results. Further experiments with bisection stimuli also showed no retrograde disruption from task B on task A. Hence, for the tasks tested here, perceptual learning does not suffer from retrograde interference.

## Introduction

**Figure 1:**
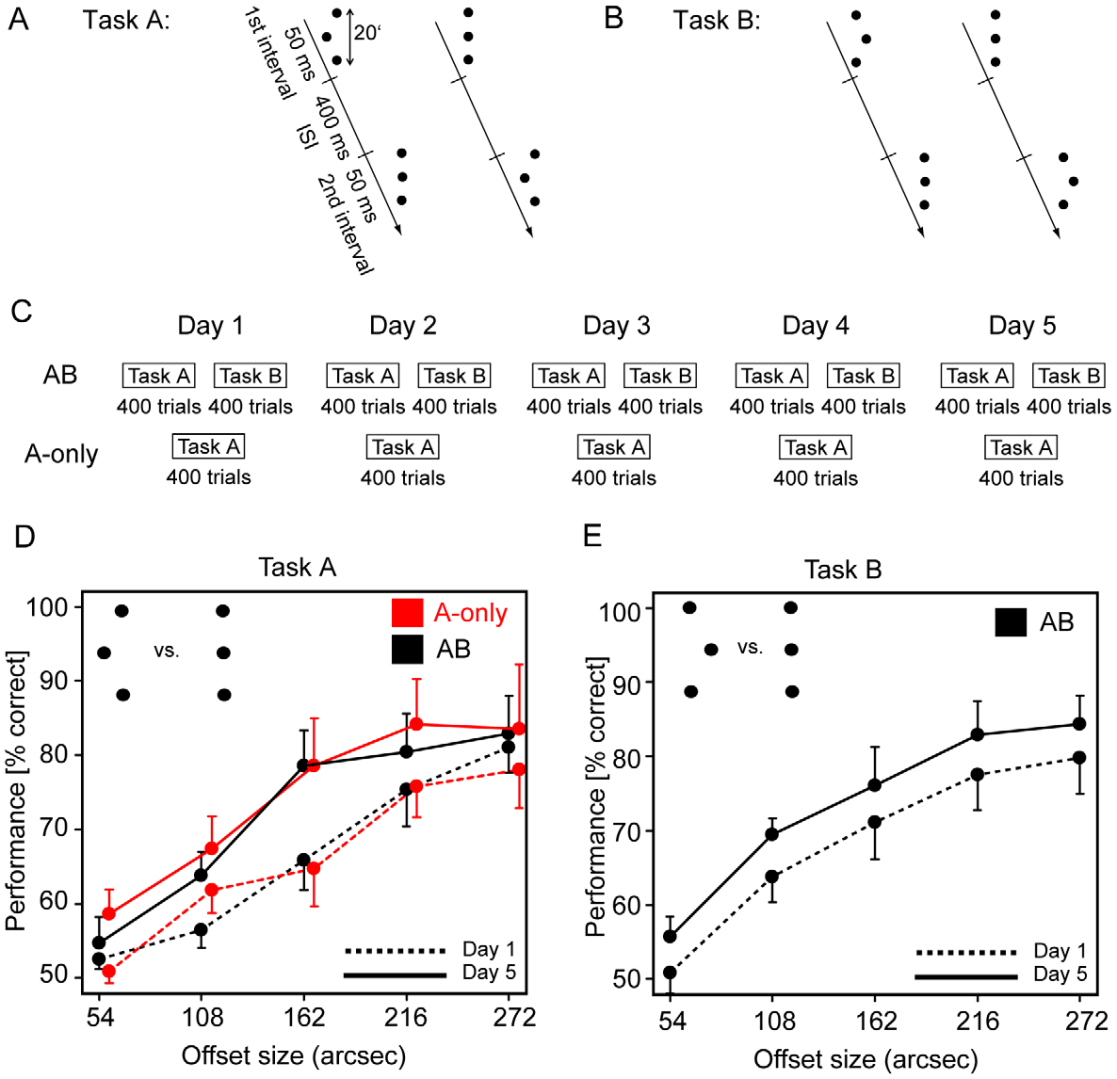
Stimuli, Procedure, and Results for the dot Vernier task. A) Task A: in each trial, participants indicated in which of two intervals a left offset dot Vernier was presented. In the other interval, an aligned dot Vernier was presented. b) Task B: in each trial, participants indicated in which of two intervals a right offset dot Vernier was presented. In the other interval, an aligned dot Vernier was presented. C) Training procedures. In the A-only group, seven participants trained 400 trials with task A on each of five days. In the AB group, seven participants trained for five days, first 400 trials with task A and immediately after 400 trials with task B. D) Results. For the A-only group, discrimination performance on task A improved from day one (red dashed line) to day five (red solid line). For the AB group, discrimination performance on task A improved in a similar fashion from day one (black dashed line) to day five (black solid line). There was no obvious difference in the improvements between the two groups. E) For the AB group, performance on task B improved from day one (dashed line) to day five (solid line). The x-axis shows the five different Verniers offsets (means ± SEM).

Many studies of perceptual learning have shown that performance strongly improves during breaks, particularly when including sleep, indicating that perceptual learning undergoes consolidation [2]–[10]. In procedural motor learning, it was shown that consolidation of a task A could be disrupted on even shorter time scales by another task B, if task B was trained directly after training with task A [11]–[16]. These results show that the improvements of task A can be disrupted by retrograde interference from task B. Likewise, it was proposed that perceptual learning can be disrupted by retrograde interference [1]. In this study [1], participants first improved discriminations of left offset dot Verniers from aligned dot Verniers (Figure 1A). Directly after, participants trained to discriminate right offset dot Verniers from aligned dot Verniers (Figure 1B). In analogy with results from motor learning, the improvement of task A was disrupted by subsequent training of task B. However, using the very same paradigm, we failed to reproduce these results. We also found no retrograde interference when two bisection stimuli were trained in separate but consecutive sessions.

## Materials and Methods

### Ethics Statement

The study was approved by the local institutional ethics committee (University of Lausanne, Switzerland).

### Participants

Forty-three naïve participants from the Ecole Polytechnique Fédérale de Lausanne (EPFL) joined the experiments after providing informed written consent. All participants had normal or corrected to normal vision as measured with the Freiburg visual acuity test [17].

### Experiment 1. Retrograde interference in a dot Vernier task

We used the very same stimuli and procedure as previously described (see [1], condition AB).

### Apparatus & Stimuli

Dot Verniers (Figure 1A,B) were presented on a black background on a 19 inch computer screen. The room was dimly illuminated (0.5 lux). Stimuli had a luminance of 82 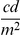 and the background luminance of the screen was 1.1 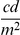.

Dot Verniers consisted of three dots with a radius of 2′ (arc min) and with a distance between the upper and lower dot of 20′ (Figure 1A). For aligned dot Verniers, the central dot was not offset, while for offset dot Verniers, the central dot was offset either to the left or to the right. We used five different offset sizes of 54, 108, 162, 216, and 270″ (arc sec). Viewing distance was 1.5 m.

### Procedure

Fourteen participants took part in Experiment 1. At the start of each trial, participants fixated a central dot for 300 ms, which flashed to indicate the presentation of two dot Verniers presented in the right lower visual field (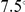 arc). Each dot Vernier was presented for 50 ms, separated by an inter-stimulus interval (ISI) of 400 ms (Figure 1A). One dot Vernier was presented without an offset (aligned dot Vernier) while the other dot Vernier was offset either to the left for task A (Figure 1A) or to the right for task B (Figure 1B). Following presentation of the second Vernier, participants had two seconds to indicate in which interval the offset dot Vernier was presented by pressing one of two buttons. Feedback was provided by changing the color of the fixation dot (green for correct and red for incorrect).

Five different offset sizes were used in the experiment: 54, 108, 162, 216, and 270″ (arc sec). During the experiment, each offset size was presented for 20 consecutive trials before changing to another offset size. At each change, participants could rest their eyes. Each offset size was presented for 80 trials in one session (5 offset sizes * 80 trials = 400 trials per session). The order of offset sizes was determined randomly. The experiment consisted of five sessions performed on five consecutive days.

Each day, seven participants first trained 400 trials with task A immediately followed by 400 trials with task B (condition AB; Figure 1C). Seven other participants, in a control group, trained 400 trials each day with task A only (condition A-only; Figure 1C).

Change in performance was determined for each task by calculating a repeated measures ANOVA with offset size (5 levels) and session (session one or five) as factors. Percent correct was used as the dependent variable.

### Experiment 2. Retrograde interference in a bisection task

Performance does not improve when bisection stimuli with different outer distances are presented interleaved trial by trial, i.e. roving [18]–[20]. For this reason, we were interested in whether there is also interference between the bisection stimuli when trained in consecutive sessions.

### Apparatus & Stimuli

**Figure 2:**
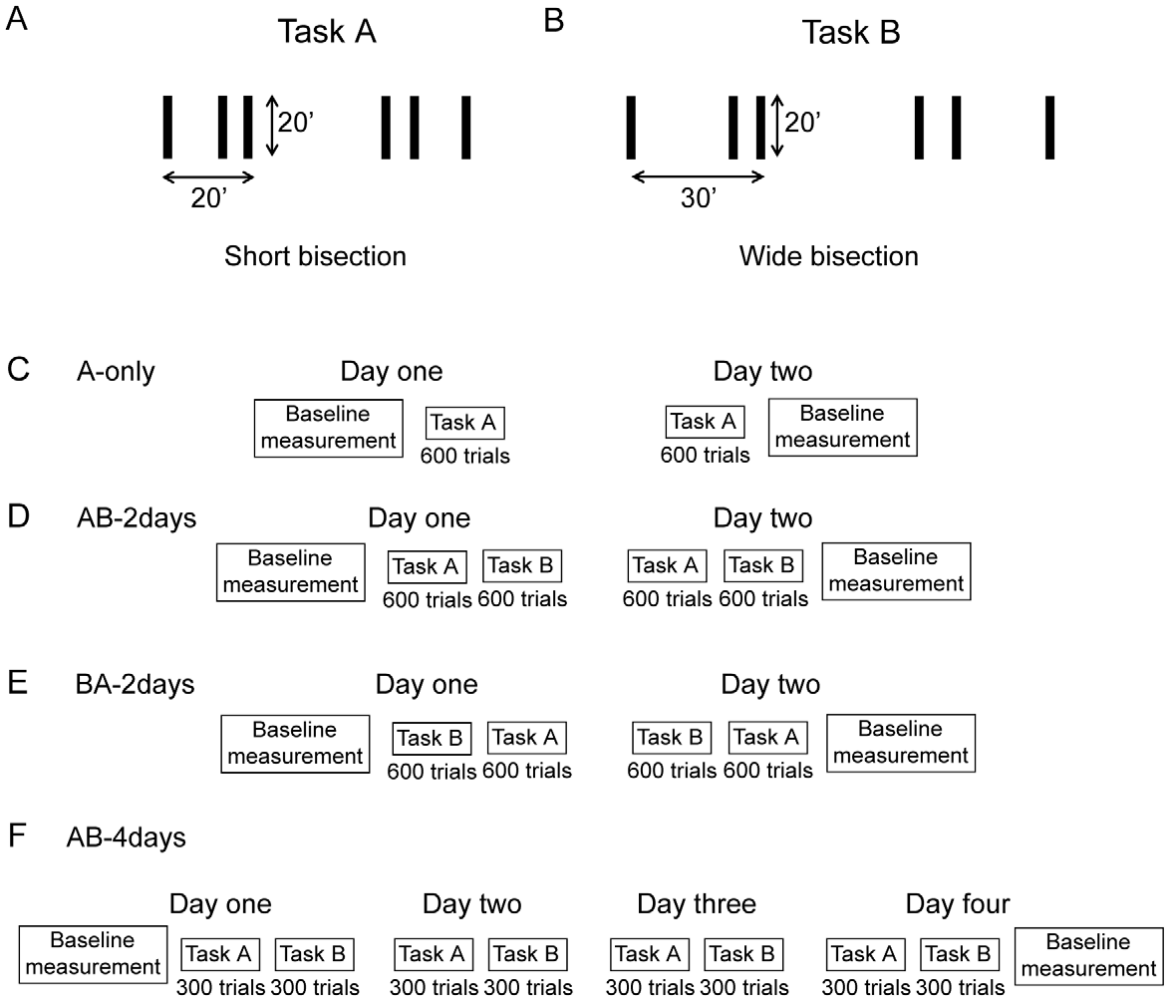
Stimuli and procedures for the bisection tasks. A) In task A, participants indicated whether the center line in a short bisection stimulus (20′ distance between the two outer lines) was offset to the left or to the right. B) In task B, participants indicated whether the center line in a wide bisection stimulus (30′ distance between the two outer lines) was offset to the left or to the right. C) Procedure for the A-only group. Baseline performance was measured on day one and two for task A for both horizontal and vertical orientations. On each of two days, participants trained 600 trials with task A, vertical orientation only. D) Procedure for the AB-2days group. Baseline performance was measured on day one and two for task A and task B for both horizontal and vertical orientations. On each of two days, participants first trained 600 trials with task A, directly followed by 600 trials with task B. Training was with the vertical orientation only. E) Procedure for the BA-2days group. Procedure was exactly the same as for the AB-2days group, except that the order of tasks was reversed, i.e. on each day, first task B, then task A, was trained. F) Procedure for the AB-4days group. Baseline performance was measured on day one and four for task A and task B for both horizontal and vertical orientations. On each of four days, participants first trained 300 trials with task A, directly followed by 300 trials with task B. During training, only vertically oriented stimuli were presented.

Bisection stimuli (Figure 2A,B) were presented on an X-Y-display (Tektronic 608) controlled by a PC via fast 16 bit D/A converters (1 MHz pixel rate). Lines were composed of dots drawn at a dot size of 250–350 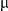
m at a dot rate of 1 MHz. The dot pitch was selected so that dots slightly overlapped, i.e., the dot size (or line width) was of the same magnitude as the dot pitch. Stimuli were refreshed at 200 Hz. Luminance of a dot grid was 80 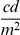 (same dot pitch and refresh rate as above) measured with a Minolta LS-100 luminance meter. The room was dimly illuminated (0.5 lux). Background luminance on the screen was below 1 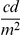.

Bisection stimuli consisted of three vertical lines of length 20′ (arcmin). For task A, the two outer lines were separated by 20′ (short bisection stimuli; Figure 2A) while for task B, this distance was 30′ (wide bisection stimuli; Figure 2B). The center line in vertical stimuli were offset either to the left or to the right. Horizontal stimuli were offset either up or down (not shown). Stimuli were presented in the fovea. Viewing distance was 2 m.

### Procedure

Twenty-nine participants joined Experiment 2. In each trial, one bisection stimulus was presented for 150 ms. Participants indicated the direction of offset for the center line by pushing one of two buttons. Auditory feedback was given for errors.

The experiment consisted of three parts. On the first day, baseline performance was determined for each task by performing two blocks of 80 trials for both vertical and horizontal orientations. Directly after, participants trained each task for two or four days (Figure 2C–F), with vertical stimulus orientations only. The training was divided into 20 blocks of 60 trials for each stimulus. At the end of the final day, baseline performance was re-measured.

During baseline measurements, the initial offset size was set to 150″. During training, the initial offset size was set to 1.5 * the mean of the two baseline measurements. A threshold was determined in each block by varying the offset size by an adaptive staircase method (PEST; [21]). A threshold for 75% correct responses was determined by maximum likelihood estimation of the parameters of the psychometric function.

Nine participants in a control group trained for two days, 600 trials per session, with task A only (A-only; Figure 2C). Seven participants in a second group trained for two days, each day first 600 trials with task A directly followed by 600 trials with task B (AB-2days; Figure 2D). Seven participants in a third group trained for two days, each day first 600 trials with task B directly followed by 600 trials with task A (BA-2days; Figure 2E). Finally, six participants in a fourth group trained for four days, each day first 300 trials with task A directly followed by 300 trials with task B (AB-4days; Figure 2F).

Change in performance for the control task was determined by comparing baseline performance before and after training. Two-tailed, paired *t*-tests were used to compare the estimated baseline thresholds. For the other three groups, change in performance was determined by repeated measures ANOVA's with factors pre/post (pre- or post-training) and task (task A or task B) as factors and baseline performance thresholds as dependent variables. Baseline performance was determined by calculating the mean of the estimated threshold in the two blocks.

## Results

### Experiment 1. Retrograde interference in a dot Vernier task

In the control experiment, seven participants trained for five sessions and 400 trials per session with task A only (condition A-only; Figure 1C). Training improved performance significantly [Figure 1D; offset size: F(4,54) = 9.09, p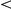.001; session: F(1,54) = 6.37, p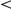.05; offset size*session: F(1,54) = 0.2, p = .92].

Seven other participants first trained 400 trials with task A and then directly after 400 trials with task B (condition AB; Figure 1C). Performance improved for both task A [Figure 1D; offset size: F(4,54) = 18.04, p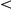.001; session: F(1,54) = 5.27, p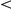.05; offset size*session: F(1,54) = 0.63, p = .65] and task B [Figure 1E; offset size: F(4,54) = 16.58, p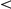.001; session: F(1,54) = 4.05, p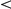.05; offset size*session: F(4,54) = 0.006, p = .99].

To determine if there was a partial disruption of task A in the AB condition, the performance of task A was compared between the AB condition and the A-only condition. A repeated measures three-way ANOVA with factors offset size (5 levels), session (one or five) and condition (A-only or AB) was calculated with percent correct as dependent variable. There were main effects of offset size F(4,108) = 24.67, p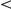.001] and session F(1,108) = 11.57, p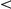.001], but no other effects nor interactions were significant (all p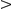.55), suggesting there was no disruption of task A in the AB condition.

Hence, we failed to replicate the result of the study by Seitz et al. [1], which found that improvements of performance for left offset dot Verniers was disrupted by subsequent training with right offset dot Verniers.

### Experiment 2. Retrograde interference in a bisection task

**Figure 3:**
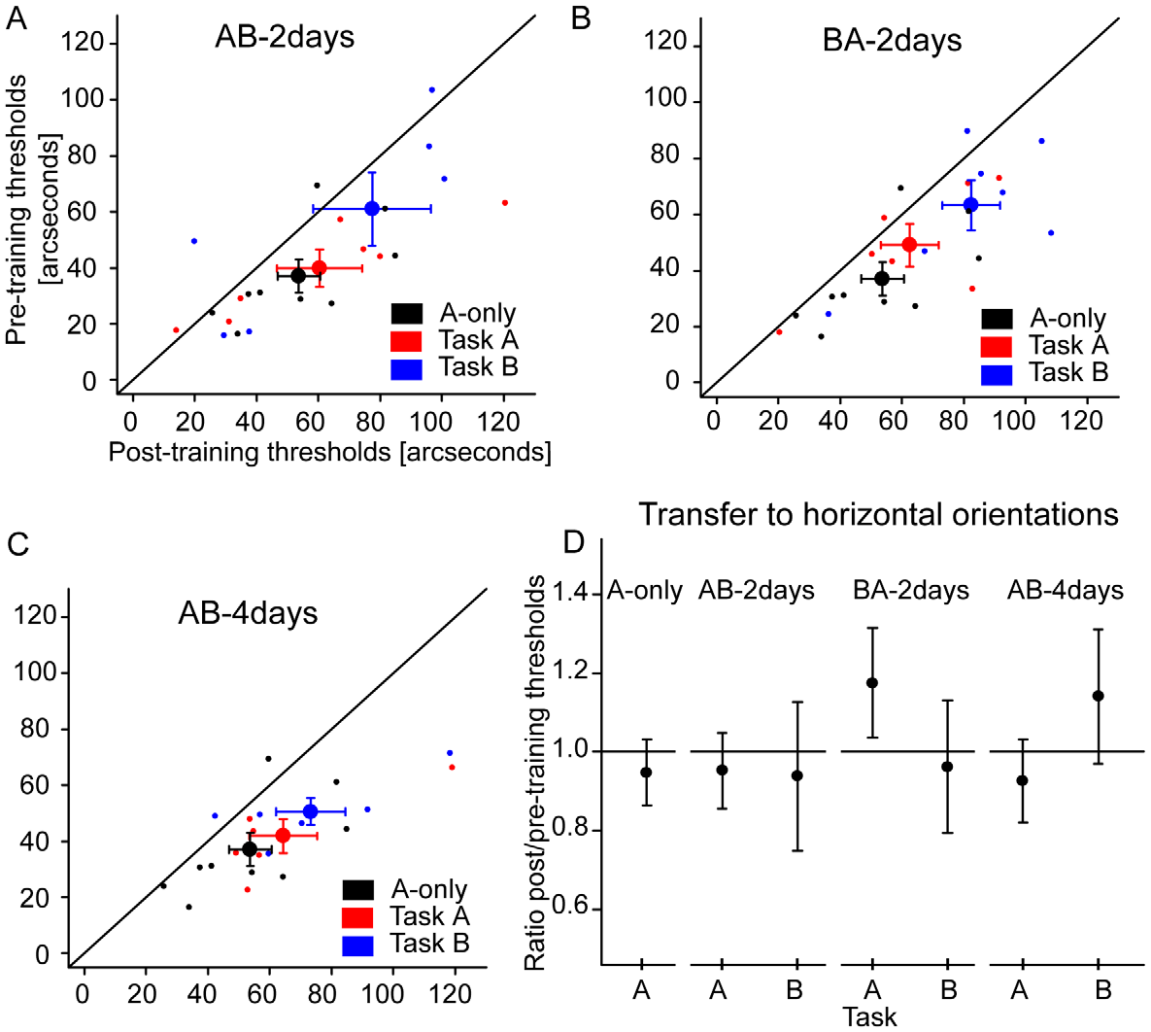
Results for the bisection task. In figures A–C, individual data are plotted as small dots while group averages are displayed as big dots (means ± SEM). Dots positioned below the diagonal black lines indicate improvement of performance. Performance for task A in the control group (A-only) is shown as black dots in all figures. * Performance improved in the A-only condition. A) Performance improved in the AB-2days condition. B) Performance improved in the BA-2days condition. C) Performance improved in the AB-4days condition. Performance for task A improved similarly in all four conditions. D) Transfer of learning was determined by calculating the ratios between post- and pre-training baseline thresholds for the untrained horizontal orientations of task A and B.. A ratio less than 1.0 indicates improvement of performance, i.e. transfer of learning. Two-tailed, one sample t-tests were used to test transfer of learning. Improvement of performance did not transfer to any untrained horizontal stimuli [A-only; task A: t(8) = −0.62, p>.55; AB-2days; task A: t(6) = −0.49, p>.65; task B: t(6) = −0.33, p>.76; BA-2days; task A: t(6) = 1.25, p>.26; task B: t(6) = −0.22, p>.83; AB-4days; task A: t(5) = −0.69, p>.52; task B: t(5) = 0.82, p>.45].

As a control, nine participants trained for two days with task A only (A-only group; Figure 2C). Performance improved with training [Figure 3A; t(8) = 3.08, p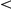.05]. There was no transfer to the untrained horizontal short bisection stimuli (Figure 3D).

Seven other participants trained for two days with task A immediately followed by task B (AB-2days group; Figure 2D). Baseline performances before and after training are shown in Figure 3B. A repeated measures ANOVA shows that performance improved with training [pre/post: F(1,18) = 7.45, p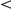.05; task: F(1,18) = 7.99, p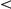.05; pre/post*task: F(1,18) = 0.10, p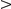.76]. This improvement was specific because there was no improvement for the horizontal untrained stimuli (Figure 3D).

Seven other participants also trained for two days, but with the order of tasks reversed, i.e. first with task B and then with task A (BA-2days group; Figure 2E). Baseline performances are shown in Figure 3C. A repeated measures ANOVA shows that performance improved [pre/post: F(1,18) = 11.75, p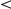.01; task: F(1,18) = 12.88, p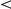.01; pre/post*task: F(1,18) = 0.35, p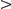.55]. There was no transfer to the untrained horizontal stimuli (Figure 3D).

While Seitz et al. [1] trained with 400 trials per session, we used 600 trials per session. Hence, there may be a possibility that consolidation occured within 600 trials of training. To test this, six new participants trained for four days with task A (300 trials) directly followed 300 trials with task B (AB-4days group; Figure 2E). Baseline performances are shown in Figure 3D. A repeated measures ANOVA indicates that performance improved [pre/post: F(1,15) = 20.31, p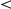.001; task: F(1,15) = 3.08, p = .10; pre/post*task: F(1,15) = 0.0007, p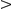.97]. Again, this improvement did not transfer to the untrained horizontal stimuli (Figure 3D).

To determine if there was any disruption of learning in the AB-2days, BA-2days, or AB-4days training groups, performance for task A was compared over the four training groups (A-only, AB-2days, BA-2days, and AB-4days). A two-way ANOVA with factors pre/post baseline thresholds (pre- or post-training) and group was conducted with performance threshold as the dependent variable. Only the effect of pre/post baseline thresholds was significant [F(1,25) = 29.15, p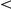.001]. There was no effect of group [F(3,25) = 0.35, p = .79] nor interaction group*pre-post training [F(3,25) = 0.34, p = .80]. These results suggest that there was no significant difference in performance for task A between the different groups and, hence, there was no disruption of perceptual learning in these conditions.

## Discussion

Long term consolidation is often important for perceptual learning. Many visual tasks often need sleep to improve performance [2]–[10]. In procedural motor learning, it has been shown that even short term consolidation of a task A can be disrupted by retrograde interference from a subsequently trained task B [11]–[16]. One influential study reported such retrograde interference also for visual perceptual learning [1]. However, using the very same paradigm, we were unable to reproduce these results (Figures 1D,E). In addition, we also found no retrograde disruption of performance with bisection stimuli (Figures 3A–C).

We do not know why the results are different between ours and the study by Seitz et al. [1] because there were only small differences in experimental design. Different screens were used, Seitz et al. used a chin rest while we did not, and while we used a dot for fixation, they used a fixation cross. Other factors may also have influenced the results, for example, slight differences in verbal instructions to the participants, which are hard to replicate exactly. Also, the participants were sampled from different populations (from different continents). Whereas we do not claim that the results of Seitz et al. [1] are not reproducable in principle, our results, which were also replicated using bisection stimuli, show that retrograde interference is not a robust effect in perceptual learning (for example, retrograde interference should not depend on whether or not a chin rest was used). In addition, we like to point out that Seitz et al. [1] did not find complete disruption of learning; participants improved performance for the offset sizes of 3.6′ and 4.5′ (see [1], condition AB).

A recent study reported retrograde interference in a texture discrimination task [22]. In this study, participants trained to discriminate the orientation of pop-out elements embedded in two different textures, A and B. Performance improved if participants trained with texture A only. However, there was no significant improvement if participants first trained with texture A and then directly after with texture B (see [22], condition Background A). This was taken as evidence for retrograde interference from training with texture B on the learning of texture A. In addition, there was proactive interference from training with texture A on the learning of texture B, which was even stronger than the retrograde interference (see [22], condition Background B). Surprisingly, performance was not disrupted when textures A and B were presented randomly interleaved trialwise (see [22], condition BGmix). These results contrast with the present study because, first, learning was not disrupted by sessionwise training, and second, in our previous studies, roving disrupted the learning [18]–[20]. Another difference between the tasks is that texture discrimination often needs sleep to improve performance [2], [3], [5]–[9] while performance in a bisection task improves already within a session [20], [23]. One reason for these discrepancies may be the complexity of the tasks. For example, texture discrimination requires participants to perform dual tasks with a backward masked target, putting heavy loads on both temporal and attentional aspects, while a bisection discrimination is a simple binary task. Therefore, interference in perceptual learning may be idiosyncratically sensitive to factors such as, for example, the presentation regime and sleep.

Why is perceptual learning possible when interfering stimuli are presented in separate sessions, but not when presented randomly interleaved trial-by-trial, in so called roving conditions? Interestingly, in contrast discrimination tasks [24]–[26], learning was possible under roving conditions if the presentation of a stimulus was preceded by a cue, indicating which stimulus alternative would be presented [26]. Performance also improved when stimuli were presented in alternated sequences, for example, A-B-A-B-…-A-B-A-B [25]. In both of these conditions, the stimuli were predictable, and it was suggested that predictability is a pre-requisite for perceptual learning [26]. The present study showed that there was no disruption when task A was presented in separate sessions from task B, arguably because each stimulus was predictable (Figures 3A–C). However, predictability does not always enable learning, for example, presenting bisection stimuli A and B in alternated sequences (A-B-A-B-…-A-B-A-B) did not improve performance [18]. Hence, although predictability may have prevented interference in contrast discrimination tasks, it does not satisfactorily explain the results in the present study.

We previously tested if perceptual learning was possible if trials with bisection stimuli were clustered, for example, A-A-A-B-A-A-A-B or A-A-A-A-A-A-B. The learning was still disrupted when up to six stimuli were clustered [18]. In the present study, perceptual learning was not disrupted when stimuli were presented in clusters of 300 and 600 trials (Figures 3A–C). Therefore, we like to speculate that consolidation of task A occured within a training session.
